# Association between Parent and Child Dietary Sodium and Potassium Intakes as Assessed by 24-h Urinary Excretion

**DOI:** 10.3390/nu8040191

**Published:** 2016-04-01

**Authors:** Carrie Service, Carley Grimes, Lynn Riddell, Feng He, Karen Campbell, Caryl Nowson

**Affiliations:** 1 Centre for Physical Activity and Nutrition Research, School of Exercise and Nutrition Sciences, Deakin University, 221 Burwood Highway, Burwood, Victoria 3125, Australia; c.service@deakin.edu.au (C.S.); lynn.riddell@deakin.edu.au (L.R.); karen.campbell@deakin.edu.au (K.C.); caryl.nowson@deakin.edu.au (C.N.); 2 Centre for Environmental and Preventative Medicine, Wolfson Institute of Preventative Medicine, Queen Mary University of London, Charterhouse Square, London EC1M 6BQ, UK; f.he@qmul.ac.uk

**Keywords:** Australia, dietary salt, parent-child, urinary sodium, nutrient, dietary potassium

## Abstract

The aim of this study was to assess the association between parent and child sodium (Na) and potassium (K) intake as assessed by 24-h urinary excretion (24hUE). Primary school children and their parent(s) provided one 24-h urine sample and information on cooking and children’s discretionary salt use. Valid urine samples were provided by 108 mothers (mean age 41.8 (5.1) (SD) years, Na 120 (45) mmol/day) (7.0 g/day salt equivalent) and 40 fathers (44.4 (4.9) years, Na 152 (49) mmol/day (8.9 g/day salt), and 168 offspring (51.8% male, age 9.1 (2.0) years, Na 101 (47) mmol/day (5.9 g/day salt). When adjusted for parental age, child age and gender a 17 mmol/day Na (1 g/day salt) increase in mother’s 24hUE was associated with a 3.4 mmol/day Na (0.2 g/day salt) increase in child’s salt 24hUE (*p* = 0.04) with no association observed between father and child. Sixty-seven percent of parents added salt during cooking and 37% of children added salt at the table. Children who reported adding table salt had higher urinary excretion than those who did not (*p* = 0.01). The association between mother and child Na intake may relate to the consumption of similar foods and highlights the importance of the home environment in influencing total dietary sodium intake.

## 1. Introduction

There is extensive evidence documenting the association between high dietary sodium (Na), low dietary potassium (K) and cardiovascular disease (CVD) risk in adults [1,2,3]. Similar to adults, Na intake has been positively, and K inversely associated with blood pressure in childhood [4,5,6,7]. Elevated blood pressure in childhood is a predisposing risk factor for hypertension in adulthood [8,9], increasing the risk of early CVD development [10].

CVD risk factors are known to cluster within families [11] and while it is recognised that parents influence their children’s dietary intakes [12], and that mothers and children’s dietary patterns are similar from an early age [13], there are few studies investigating the association between parent and child’s Na and K intakes. The aim of this study were to assess the association between parent and child sodium (Na) and potassium (K) intake as measured by 24-h urinary excretion (24hUE) in primary schoolchildren.

## 2. Materials and Methods

### 2.1. Participants

A cross-sectional study, Salt and Other Nutrient Intakes in Children (SONIC) was conducted in Victoria, Australia from June 2010 to May 2013. Full details of the SONIC study design, procedures and protocols have been published elsewhere [14]. This analysis included children (including sibling pairs and trios) (*n* = 191) who had a parent (*n* = 171) complete a 24-h urine collection. Written consent was obtained from all participating children, as well as their parent/guardian. Ethics approval was granted by the Deakin Human Research Ethics Committee (Project ID: EC-62-2009). Permission to enter government schools was granted by the Victorian Department of Education and Early Childhood Development (2011_001151).

### 2.2. Data Collection and Measures

#### 2.2.1. Demographic Characteristics

Information on age and gender were obtained using a questionnaire completed by parents. Height and weight of the children was measured on site by trained researchers using standard protocols [14]. Body mass index (BMI) was calculated as body weight (kg) divided by the square of body height (m^2^). Weight status of children was defined by International Obesity Taskforce (IOTF) BMI reference cutoffs [15,16].

#### 2.2.2. 24-h Urine Collection

Participants could opt to complete the 24-h urine collection on a school day or non-school day. All were instructed to commence the collection at any suitable time and followed the usual 24-h urine protocol by emptying their bladder, discarding this urine, and recording this as the start time [14]. Following this all urine was collected until the corresponding 24-h finish time.

### 2.3. Urine Analyses

Urinary Na and K concentration were assessed by a commercial laboratory on the Siemens Advia 2400 analyser (Dorevitch Pathology, Melbourne, Australia) using indirect ion selective electrodes whereas urinary creatinine was assessed using the Jaffe Reaction [17]. The coefficient of variation (CV) for sodium and potassium was less than 1% and for creatinine it was 3.25%.

### 2.4. Potential under/over Collections: 24-h Urine Samples

Of the 191 child participants, 190 completed a 24 h urine collection. Where urine collections were not exactly 24 h in duration (but within 20–28 h) values of urinary sodium, potassium, creatinine and total volume were standardised to a 24-h period. Four criteria including collection time, minimum volume, minimum creatinine excreted and if the participant reported missing >1 void [16] were used to determine the completeness of urine samples. In total, 15 of the children’s collections were excluded on the basis of this criteria and a further two collections were excluded due to laboratory processing errors.

Five collections were excluded from parents: <20 h or >28 h (*n* = 5), 24-h urinary creatinine excretion was <4 mmol for females or <6 mmol for males (*n* = 1). No parent reported missing a collection and all volumes were greater than 500 mL [18]. This left a total analytical sample of 168 children and 148 parents. Based on these, the corresponding parent or child to those who were excluded were subsequently also excluded (children *n* = 5, parent *n* = 17).

#### Discretionary Salt Use

Both parents and children reported on the frequency of discretionary salt use. For parents three questions were included on a questionnaire, “Do you add salt during cooking?” [19]; “Do you place a salt shaker on your table at meal times?” and “Does your child add salt to their meal at the table or sandwich preparation?” Children were also separately asked “Do you add salt to your meal at the table?” [19]. For all questions participants could respond “yes, usually”, “yes, sometimes”, “no” or “don’t know”.

Parents reported on each of these three questions for each child, however for the questions: “Do you add salt during cooking?” and “Do you place a salt shaker on your table at meal times?” findings are reported only at the family level, *i.e.*, in instances where the parent had more than one child participate the parental responses for both questions were crosschecked to determine if the parent had reported the same response for each child. Only in one instance did a parent report a different response for each child for the question related to placement of a salt shaker on the table at meal times, this data was excluded.

### 2.5. Statistical Analysis

Statistical analyses were conducted using statistical software package STATA/SE 13. A *P* value of <0.05 was considered significant. The molecular weight of sodium chloride (58.5 mmol/g) was used to convert sodium (mmol/day) to the salt equivalent (g/day) (*i.e.*, ((Na mmol × 58.5)/1000) [20]. Descriptive statistics (mean (SD) (SEM)) and (frequency % (*n*)) are reported for continuous and categorical variables, respectively. Linear regression, which allows for adjustment of school cluster, was used to compare differences in electrolyte intake across sexes. To assess the association between child urinary electrolyte excretion and parent urinary electrolyte excretion multiple linear regression was used. Regression models were stratified by parental gender and unadjusted and adjusted models are presented. Model A was adjusted for parental age, and child’s age and gender with model B adjusted for model A in addition to parent’s reported use of cooking salt. Model C was adjusted for model B plus adjustment for child’s reported use of table salt. In 21 instances the child had both a mother and father in the study and were included in both models. Children from the same family *n* = 64 (26 sibling pairs and 4 sibling trios), 38% of sample) were included in the analysis, and hence in these instances the parental data was within the regression model twice (for sibling pairs) or three times (for sibling trios). To account for clustering of students within schools clustered robust standard errors were used.

Linear regression was used to assess the association between discretionary salt use behaviours and child’s urinary sodium excretion. For this analysis, the response categories “yes, usually” and “yes, sometimes” were collapsed together creating a dichotomous variable of “yes, usually or sometimes” *vs*. “no”. All models were adjusted for school cluster, child’s age, gender and day of urine collection.

## 3. Results

One hundred and sixty eight children from 134 families (104 single child families, 26 sibling pairs, 4 sibling trios) and 148 parents (108 mothers and 40 fathers) provided a valid 24hUE (Table 1). Just over half of the children were male with approximately two-thirds (*n* = 114, 68%) of participants completing the 24-h urine collection on a weekend day (Table 2). The majority of parents completed the urine collection on a non-school day (*n* = 109, 77%). Seventy eight percent of parent-child pairs provided urine samples on the same day. Table 1 shows urinary excretion of electrolytes in children. There were no differences in mean Na intake or Na:K ratio between boys and girls (Na: 108.0 (SD 46.2)) mmol/day *vs*. 92.8 (SD 46.2) mmol/day, *p* = 0.06) (Na:K ratio:2.4 (SD 1.2) *vs*. 2.2 (SD 1.0), *p* = 0.414). Comparatively, mean K excretion was significantly higher in boys (51.0 (SD 19.3) mmol/day) than girls (44.1 (SD 15.9) mmol/day) (*p* = 0.01).

Electrolyte excretion of parents is presented in Table 2. Males had significantly higher intakes of Na (152.2 (SD 49) mmol/day *vs*. 120.0 (SD 45) mmol/day, *p* = 0.002)) and K (91.1 (SD 40) mmol/day *vs*. 67.8 (SD 19) mmol/day, *p* < 0.001) compared to females. There was no gender difference in the mean Na:K ratio (1.9 (SD 0.8) *vs*. 1.9 (SD 0.8), *p* = 0.984).

A 1 g/day increase in mother’s salt intake was associated with a 0.20 g/day increase in child’s salt intake (adjusted for child’s age, gender and parental age), (*p* = 0.04), this association remained after further adjustment for both parent cooking salt and child table salt use (Model C, Table 3). There was no association between mother and child’s K excretion or father and child’s Na or K excretion.

**Figure 1:**
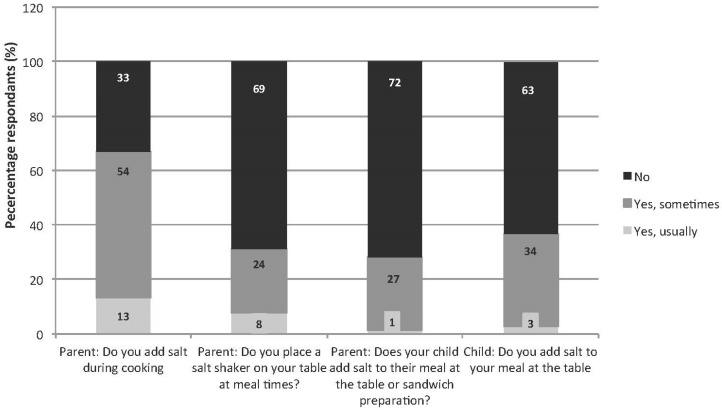
Discretionary salt use by children and parents (frequency %).

The majority of parents (67%) reported adding salt to cooking either “yes, usually” or “yes, sometimes”, whereas most (69%) indicated they did not provide a salt shaker on the table at meal times (Figure 1). Thirty-seven percent of children reported adding salt at the table either “yes, usually” or “yes, sometimes”. Comparatively the proportion of parents who reported that their child added salt at the table either “yes, usually” or “yes, sometimes” was lower (28%) (*p* < 0.001). Mean Na excretion was higher in those children who reported adding salt at the table (112.1 *(*SD 49.8*)* mmol/day), compared to those children reported not adding salt at the table (94.3 *(*44.1*)* mmol/day) (*p* = 0.01). When adjusted for age, gender and day of urine collection sodium excretion was 16.8 (SEM 6.9) mmol/day (*p* = 0.02) higher in those children who reported adding table salt. Conversely, mean Na excretion was lower in those children whose parents reported adding salt to cooking (94.8 *(*SD 45.2) mmol/day compared to those who reported not adding salt during cooking (111.2 (47.8) mmol/day (*p* = 0.02). Similarly, after adjustment for age, gender and day of urine collection this difference remained, whereby sodium excretion was 17.3 (SEM 6.5) mmol/day lower, *p* = 0.01 in children whose parents reported adding salt during cooking. There was no difference in sodium excretion of children whose parents reported placing a salt shaker on the table (data not shown).

## 4. Discussion

In this sample of Australian schoolchildren a significant association was observed between mother and child’s Na intakes. This association remained, after adjustment for discretionary salt use, indicating the relationship between child and mother sodium intakes is explained by similarities in the consumption of processed foods. There was no association between father and child’s Na intake, and also no association between either parent and child K intakes. This finding is similar to the Framingham Heart Study (FHS) which using three day food diaries found a correlation between mother and child’s Na intake (*r* = 0.30, *p* < 0.001), but not father’s Na or either parents K intakes [21]. Mother’s nutrient intakes may be expected to have a stronger correlation to the intake of their children compared with fathers due to higher maternal involvement in the provision of food particularly during early childhood years [22,23]. According to national data, major sources of Na in childrens’ and adults’ diets are similar: cereal-based products (adults 24%, children 30%), and cereal products (adults 18%, children 21%), followed by meat, poultry and game (adults 19%, children 17%) and milk products (adults 8%, children 9%); therefore, we would expect there to be a relationship between parent and child sodium intake [24].

Unlike Na, the major dietary sources of K differ between adults and children [24]. Adults obtain the majority of K intake from vegetables and vegetable products (adults 18%, children 15%) whereas children obtain K mostly from milk and milk products (adults 12%, children 19%) which may explain the lack of association between urinary K in parents and children [24].

With regards to discretionary salt use, the use of salt by parents during cooking was more common (67%) that the use of salt at the table (32%) by the child. It was found that the child was more likely to report that they added salt at the table than if the parent reported on the child’s behalf. This may be because children are less inclined to provide socially desirable responses compared with parents [25]. We found that those children who reported usually or sometimes adding salt at the table had a higher Na intake. This indicates that although it is acknowledged that the major sources of Na are processed foods [26], a child’s use of table salt also contributes to overall Na intake and that advice to avoid adding salt at the table could assist in reducing total dietary Na intake. It is unknown why parent’s reported use of cooking salt was found to be linked to a lower Na intake in children however it may be that parents who add salt to cooking may use less processed foods and therefore their children are exposed to lower amounts of overall sodium.

While studies have indicated parents can directly influence the dietary choices of children [12,27], other factors such as environmental influences, siblings and school policies are also likely to be important in influencing children’s nutrient intakes [21]. The importance of interventions engaging and supporting parents in the home environment to promote healthy behaviours in children has been noted in previous research [28,29]. This is largely due to family members consuming similar foods and parents playing a direct role in children’s eating via their behaviours, attitude and feeding styles [23,30,31].

Taste preferences for Na are known to be determined by exposure to salty foods and a lifelong exposure to high Na intakes may lead to the development of a preference for high Na foods [32]. It has also been demonstrated that short-term exposure to Na is likely to increase preference for salty foods and subsequently liking and consumption of salty foods in children [33,34]. Given that taste and dietary preferences appear to develop and track over the life course [35,36], it is important that Na intake is not excessive during childhood. There is the potential for mothers and fathers to play an important role in the reduction in children’s salt intake, by choosing lower salt manufactured products and removing the salt shaker from the table thereby reducing a salt taste preference early in life. 

A major strength of this study is the use of an objective measure of Na and K intake [37,38]. Urine excretion of dietary Na has been found to be as high as 95%–98% whereas urinary K represents 85%–89% of dietary intake [39]. It should be acknowledged that 22% of child and parent urines were collected on different days which is a limitation and observed associations (or lack of) may have been impacted by the large intra-individual daily variation in Na excretion [39]. Sodium intake has been shown to be higher amongst Australian children with a low socioeconomic background compared to those from a high socioeconomic background [40]. In the current study participants were drawn from a convenience sample of children who were of a relatively high socioeconomic background [41], as such this limits the generalizability of the findings across broader soceioeconomic groups. Furthermore, the study was limited by a low response rate [14] and parental self-selection for inclusion. It is also possible that participants had an interest in health and hence may be different from the general population.

## 5. Conclusions

In this sample of primary schoolchildren, there was an association between mother and child Na intakes. Children who reported adding table salt had higher urinary excretion than those who did not, highlighting an important target for salt reduction interventions. It is important whilst continuing to endorse initiatives to reduce the amount of salt in the food supply as the association between mother and child’s sodium excretion appears to be due to similar food consumption rather than discretionary salt use.

Policies and strategies that target both children and their parents are likely to assist in developing lifelong eating habits that minimize discretionary table salt and ensure optimal fruit and vegetable intake that will assist in reducing the risk of CVD later in life.

## Figures and Tables

**Table 1:** Demographic characteristics, anthropometry and 24-h urine collection results by age-group (*n* = 168).

Measure	Total (*n* = 168)		4–8 Years (*n* = 76)		9–12 Years (*n* = 92)	
	Mean or *n*	SD or %	Mean or *n*	SD or %	Mean or *n*	SD or %
Gender (male) ^1^	87	51.8%	41	54.0%	46	50.0%
Age (years) ^2^	9.1	2.0	7.3	1.1	10.6	1.0
BMI ^1,3^						
Underweight	18	10.7%	12	15.8%	6	6.5%
Healthy weight	129	76.8%	61	80.3%	68	73.9%
Overweight	17	10.1%	2	2.6%	15	16.3%
Obese	4	2.4%	1	1.3%	3	3.3%
Day of urine collection ^1^						
School day	54	32.1%	23	30.3%	31	33.7%
Non-school day	114	67.9%	53	69.7%	61	66.3%
Na (mmol/24-h) ^2^	101	47	90	36	110	53
Salt equivalent (g/24-h) ^2^	5.9	2.7	5.2	2.1	6.4	3.1
K (mmol/24-h) ^2^	48	18	43	15	52	20
Na:K ^2,4^	2.3	1.1	2.3	1.1	2.3	1.2
Creatinine (mmol/24-h) ^2^	5.4	2.0	4.3	1.3	6.3	2.0
Total volume (mL) ^2^	858	414	730	329	961	449

^1^ n (%); ^2^ mean (SD); ^3^ Based on IOTF BMI reference cut offs [15,16]; ^4^ Molar ratio.

**Table 2:** Demographic characteristics and 24-h urine collection of parents of schoolchildren by gender (*n* = 148).

Measures	Female (*n* = 108) 73.0%		Male (*n* = 40) 27.0%	
	Mean or *n*	SD or %	Mean or *n*	SD or %
Age (years) ^1,3^	41.8	5.1	44.4	4.9
Day of urine collection ^2^				
School day	26	25.0%	6	16.2%
Non-school day	78	75.0%	31	83.8%
Na (mmol/24-h) ^1^	120	45	152	49
Salt equivalent (g/24-h) ^1^	7.0	2.6	8.9	2.9
K (mmol/24-h) ^1^	68	19	91	40
Na:K ^2,4^	1.9	0.8	1.9	0.8
Creatinine (mmol/24-h) ^1^	10.0	2.2	16.0	6.2
Volume (mL) ^1^	1890	758	2175	1033

^1^ mean (SD); ^2^ *n* (%); ^3^ Age missing for 8 mothers and 3 fathers; ^4^ Molar ratio.

**Table 3:** Association between parent-child sodium and potassium excretion.

	Adjusted *R*^2^ (*p* Value)	B	95% CI	*p* Value
Mothers (*n* = 138 mother-child pairs)				
Salt equivalent (g)				
Unadjusted model	0.04 (0.08)	0.18	(−0.02, 0.39)	0.08
Model A ^1^	0.16 (0.001)	0.20	(0.01, 0.40)	0.04
Model B ^2^	0.19 (<0.001)	0.18	(−0.01, 0.38)	0.06
Model C ^3,4^	0.22(<0.001)	0.21	(0.02, 0.41)	0.04
Fathers (*n* = 51 father-child pairs)				
Salt equivalent (g)				
Unadjusted model	0.04 (0.22)	0.22	(−0.14, 0.57)	0.22
Model A ^1^	0.16 (0.02)	0.21	(−0.13, 0.55)	0.21
Model B ^2^	0.17 (0.03)	0.19	(−0.14, 0.52)	0.25
Model C ^3^	0.20 (0.03)	0.14	(−0.23, 0.50)	0.45
Mothers (*n* = 138 mother-child pairs)				
Potassium (mmol)				
Unadjusted model	0.004 (0.42)	0.05	(−0.01, 0.19)	0.42
Model A ^1^	0.24 (<0.001)	0.12	(−0.01, 0.25)	0.08
Fathers (*n* = 51 father-child pairs)				
Potassium (mmol)				
Unadjusted model	0.01 (0.37)	0.05	(−0.1, 0.18)	0.37
Model A ^1^	0.13 (0.07)	0.10	(−0.06, 0.27)	0.22

^1^ Model A: Adjusted for parental age, child’s age and gender. Excludes *n* = 8 mothers and 4 fathers without age; ^2^ Model B: Model A plus adjustment for parental reported use of cooking salt; ^3^ Model C: Model B plus adjustment for child’s reported use of table salt; ^4^ Excludes *n* = 2 children with missing data on table salt use.

## Abbreviations

The following abbreviations are used in this manuscript:

24hUE	24-h urinary excretion
BMI	body mass index
CV	coefficient of variation
CVD	cardiovascular disease
IOFT	International Obesity Taskforce
K	potassium
Na	sodium
SD	standard deviation
SEM	standard error of the mean
SONIC	Salt and Other Nutrient Intakes in Children
